# Development of mycelium-reinforced dialdehyde cellulose–polyvinyl alcohol composites for emerging high-performance sustainable materials

**DOI:** 10.1016/j.synbio.2026.03.012

**Published:** 2026-03-27

**Authors:** Pattaravaree Petchsong, Nattawut Boonyuen, Papichaya Kwantong, Salilaporn Nuankaew, Charuwan Chuaseeharonnachai, Sarute Ummartyotin

**Affiliations:** a Department of Materials and Textile Technology, Faculty of Science and Technology, Thammasat University, Pathum Thani, 12120, Thailand; b Thammasat University Center of Excellence in Smart Materials, Energy, Biochemistry, Food Technology and Textile Innovation for Sustainable Environment, Thammasat University, Pathum Thani, Thailand; c Center of Excellence on Petrochemical and Materials Technology, Chulalongkorn University, Soi Chula 12, Phayathai Road, Pathumwan, Bangkok, 10330, Thailand; d National Center for Genetic Engineering and Biotechnology (BIOTEC), National Science and Technology Development Agency (NSTDA), Pathum Thani, 12120, Thailand

**Keywords:** Mycelium, Dialdehyde cellulose, Polyvinyl alcohol, Bio-composites, *Ganoderma boninense*, Sustainable materials

## Abstract

Dialdehyde cellulose (DAC), featuring reactive carbonyl groups introduced via oxidation of hemp-derived cellulose fibers (confirmed by FTIR spectroscopy), was synthesized to promote strong hydrogen bonding with polyvinyl alcohol (PVA) for composite development. In this study, DAC was incorporated into hydrogel composites reinforced with mycelium from three fungal species: *Ganoderma boninense*, *Pleurotus eryngii*, and *P. ostreatus*, thereby enabling direct comparison of their effects on composite performance. Notably, this work pioneers the use of *G. boninense*—a phytopathogenic fungus with a dense, hydrophobic mycelial architecture (contact angle 104.1°)—as a novel reinforcing agent in the DAC–PVA matrix. The resulting mycelium-reinforced composites demonstrated enhanced thermal stability, as confirmed by thermogravimetric analysis (TGA), which identified a multi-stage pyrolysis process primarily occurring between 200 and 500 °C. Mechanical characterization further revealed significant improvements in tensile strength and elongation at break: *P. eryngii* reinforcement yielded the highest tensile strength (18.3 MPa). At the same time, *G. boninense* imparted exceptional flexibility (elongation at break 93.3%). Furthermore, the composites exhibited significantly reduced swelling ratios—*Ganoderma boninense*, *Pleurotus eryngii*, and *P. ostreatus* recorded swelling ratios of 400.9%, 405.3%, and 319.3%, respectively—accompanied by enhanced dimensional stability and a transition from hydrophilic to hydrophobic surfaces, as confirmed by contact angle measurements. Altogether, these mycelium-reinforced DAC–PVA composites represent promising sustainable materials, suitable for diverse applications that require superior mechanical performance, water resistance, and thermal stability, such as biodegradable packaging, biomedical scaffolds, environmental sensors, and construction.

## Highlights

• Dialdehyde cellulose was synthesized from hemp using an alkaline treatment technique.
• Mycelium-based composites were developed by dialdehyde cellulose, PVA and fungal species.
• Three species — *Ganoderma boninense*, *Pleurotus eryngii,* and *P. ostreatus* were employed.
• The incorporation of mycelium significantly enhanced the mechanical properties.
• Mycelium-reinforced composites exhibited thermal stability and swelling behavior.

## Graphical abstract

**Figure:**
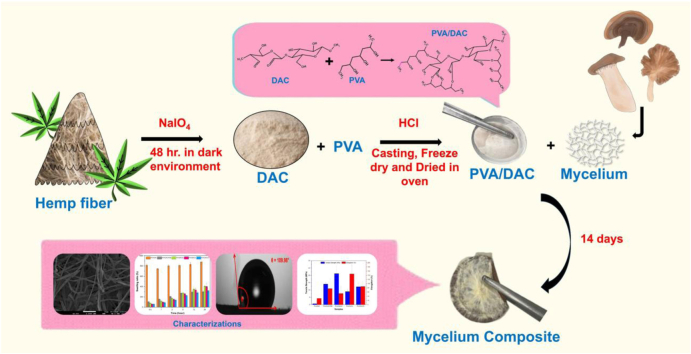

## 1 Introduction

In recent years, rapid global population growth has spurred extensive industrial development to facilitate daily life. While these technologies offer significant engineering benefits, they have also raised concerns regarding landfill accumulation and end-of-life material disposal. Consequently, clean technology has been increasingly employed to support waste management and mitigate environmental pollution [1]. Clean technology, also known as “climatetech,” encompasses processes and products that reduce negative environmental impacts by improving energy efficiency and promoting sustainable resource utilization. This field includes recycling, renewable energy, green transportation, logistics, and green chemistry [2,3]. A significant area within clean technology is the development of functional materials for diverse applications such as automotive parts, construction components, active food packaging, medical technologies, and electronic devices [4,5]. These technologies provide numerous benefits, including reduced carbon footprints, decreased energy consumption and processing costs, improved biodegradability, and greater alignment with eco-friendly practices.

Cellulose, with the empirical formula (C_6_H_10_O_5_)_n_, remains one of the most abundant naturally occurring bio-based materials. This polysaccharide consists of a linear chain of β(1→4) linked d-glucose units [[6], [7], [8]]. As a predominant component of plant cell walls, and also found in some bacteria and algae, cellulose can be extracted from various biomass feedstocks like hemp [9], water hyacinth [10], pineapple leaves [11], coconut husks [12], and durian peels [13]. Its abundant availability and favorable structural properties make it ideal for sustainable material development. Purified cellulose serves as a foundational material in bio-based composites, enhancing their mechanical strength and biodegradability. Technically, cellulose offers high stiffness, substantial chemical resistance, and excellent thermal stability, rendering it inattractive for many practical applications. For example, Sirichaibhinyo et al. (2024) extracted microcrystalline cellulose from hemp, enhancing the mechanical and electrochemical performance of separator-membrane composites for lithium-ion batteries [14]. Likewise, Promdontree et al. (2024) produced cellulose nanocrystals from hemp to improve the injectability and mechanical properties of thermo-responsive injectable hydrogels [15].

Despite these advantages, cellulose alone is difficult to process into stable, water-resistant structures without a co-component, crosslinker, or chemical modification. Dialdehyde cellulose (DAC)—typically generated by periodate oxidation—introduces reactive aldehydes at C2 and C3 of the anhydroglucose (pyranose) ring. These aldehydes can form covalent linkages with nucleophilic groups (e.g., acetal/hemiacetal bonds with polyols such as PVA, or Schiff bases with amines), thereby enhancing interfacial adhesion and moisture resistance. Consequently, DAC can function both as a crosslinker and as a reinforcing cellulose phase within composite networks [[16], [17], [18]]. Polymeric composites offer a solution, enhancing material efficiency for advanced engineering contexts [16,17]. Polyvinyl alcohol (PVA), a water-soluble synthetic polymer used in papermaking, textiles, and medical devices, is an effective matrix for cellulose-based composites. PVA combined with cellulose enhances mechanical strength, dimensional stability, and thermal resistance, suitable for medical technology, food packaging, and electronic substrates. Other plastics like HDPE, LDPE, PVC, and PP also show improved properties with cellulose integration. For instance, Khouaja et al. (2021) found that increasing cellulose content in HDPE composites raised dielectric constants, loss factors, and thermal stability [18]. Hossain et al. (2023) demonstrated that 0.2 wt% jute-derived cellulose in unsaturated polyester optimized tensile strength, bending strength, elongation, and impact strength [19]. Kanbua et al. (2023) developed a cellulose/polyether block amide composite with excellent electrolyte uptake, wettability, and thermal stability for battery membrane applications [20]. Kang et al. (2024) created a cellulose-based adhesive for bamboo composites, achieving a densely cross-linked network with improved bonding strength and water resistance via covalent bonding with epoxy groups on the bamboo surface [21] and Li et al. fabricated a composite hydrogel by introducing cellulose nanofibers (CNF) and a hydrophobic system into a dual network of polyacrylamide and polyvinyl alcohol (PAM/PVA). This was done to synergistically strengthen the material, using the CNF to enhance and bridge the two polymer networks through extensive hydrogen bonding [22]. Addressing these issues, sustainability initiatives promote minimizing hazardous chemical reagents in product design and processes [23]. Developing sustainable composites is a key strategy, mitigating environmental impact while advancing high-performance materials. Among these, mycelium-based composites (MBCs) are highly effective due to their unique structural properties. Mycelium, the root-like fungal structure of branching hyphae, colonizes filler-matrix interfaces in composites, enhancing mechanical properties and adhesion. Recent studies highlight MBC benefits: Gaff et al. (2024) showed mycelium enhances tensile/flexural strength, hardness, and thermal insulation [24]. Livne et al. (2024) reported MBCs reduce embodied energy, improve CO_2_ sequestration, and offer optimal compressive strength and thermal conductivity [25]. Zhang et al. (2023) developed lightweight, thermally insulating MBCs with hierarchical porous architecture [26]. Despite these advantages, MBCs often exhibit inconsistent mechanical performance, moisture sensitivity, and inadequate thermal stability. This study specifically addresses these limitations by strategically integrating DAC and PVA with three carefully selected fungal species—*Ganoderma boninense*, *Pleurotus eryngii*, and *Pleurotus ostreatus*. Each species is chosen to provide unique structural reinforcement, enhancing the composite's mechanical robustness, hydrophobicity, and dimensional stability, thereby overcoming common barriers to wider industrial application.

In mycelium-based composites derived from *Ganoderma boninense*, *Pleurotus eryngii*, and *P. ostreatus*, interspecific variation in hydrophobin biosynthesis and surface deposition represents a key determinant of divergent hydrophobic behavior. Hydrophobins are classically characterized as cysteine-rich, amphipathic proteins that undergo spontaneous self-assembly into ordered rodlet monolayers at air–solid and air–water interfaces, substantially reducing surface free energy and modulating wettability in fungal tissues and engineered materials [[27], [28], [29], [30]]. Empirical studies on mycelium films, membranes, and fungal biopolymer interfaces demonstrate strong correlations between hydrophobin abundance, rodlet layer continuity, and water contact angle responses. This body of evidence supports the mechanistic interpretation that species producing higher hydrophobin quantities—such as *Ganoderma*—exhibit greater surface energy modification compared with *Pleurotus* species, thereby explaining the enhanced hydrophobicity observed in our DAC–PVA–mycelium composites.

From a structural perspective, *G. boninense* develops a trimitic hyphal architecture comprising generative, skeletal, and binding hyphae, resulting in a densely interwoven and mechanically robust network optimized for load distribution and polymer interfacial adhesion [31,32]. In contrast, *P. eryngii* and *P. ostreatus* possess monomitic systems composed exclusively of generative hyphae, yet differ markedly in hyphal diameter, branching complexity, and packing density. The thicker, more rigid generative hyphae of *P. eryngii* contribute to superior tensile reinforcement, whereas the finer and more intricately branched hyphae of *P. ostreatus* impart greater flexibility and balanced mechanical performance. These species-specific traits align with previous observations indicating that fungal cell wall composition, hyphal geometry, and network architecture collectively govern stiffness, moisture resistance, and failure behavior in mycelium-based materials [[33], [34], [35]].

Furthermore, distinct β-glucan profiles among these species—including differences in β-1,3/β-1,6 branching ratios and crosslinking potential—modulate composite densification, water affinity, and matrix–mycelium interfacial bonding [33]. Taken together, the integrated biochemical, morphological, and structural evidence provides a coherent and mechanistically grounded explanation for the species-dependent hydrophobicity, mechanical reinforcement, and overall performance enhancements observed in our DAC–PVA–mycelium composites.

The integration of *Ganoderma boninense*, *Pleurotus eryngii*, and *P*. *ostreatus* in this research leverages their distinct mycelial architectures to optimize composite performance. *P. eryngii* and *P. ostreatus*, well-established in MBCs, contribute robust mechanical reinforcement, combining strength with scalability. Strategically integrating these species optimizes the DAC–PVA design for improved mechanical performance, dimensional stability, and moisture resilience [[35], [36], [37], [38]], making them promise for DAC-PVA composites due to their favorable properties and scalability. Within this system, *G. boninense*, the causal agent of Basal Stem Rot in oil palm, forms a dense, structurally robust network, contributing to increased rigidity and stronger interfacial bonding. Its dense, hydrophobic mycelial architecture also substantially improves water resistance and enhances flexibility [39,40]. The use of *G. boninense* in green composite design is an innovative approach, distinct from conventional species like *P. ostreatus* and *G. lucidum*. Collectively, these fungi are expected to synergistically enhance mechanical strength, durability, and interfacial adhesion, developing high-performance, sustainable composites [[41], [42], [43]].

The distinct structural and functional contributions of *G. boninense*, *P. eryngii*, and *P. ostreatus* further justify their inclusion. *G. boninense* offers a densely interwoven, hydrophobic mycelial network that minimizes water absorption and promotes dimensional stability [39]. *P. eryngii* provides excellent tensile strength through its thick, fibrous hyphae [35], while *P. ostreatus* offers a balanced enhancement of strength and elongation, ideal for scalable applications [38]. Beyond mechanical reinforcement, all three species aim to improve water resistance, biodegradability, and thermal stability. *G. boninense* is anticipated to impart near-waterproof behavior, while *P. eryngii* and *P. ostreatus* are expected to reduce swelling ratios and equilibrium water content. Together, these fungi are intended to synergize, producing composites that are mechanically robust, flexible, and environmentally sustainable, addressing key demands in clean material innovation [25,26,44]. While MBC studies often use *Pleurotus ostreatus* and *Ganoderma lucidum*, the phytopathogen *G. boninense* remains largely unexplored in material science. This study introduces *G. boninense* as a novel reinforcing agent in the DAC–PVA matrix, leveraging its dense, hydrophobic hyphal architecture to enhance elongation, water resistance, and structural flexibility, thereby expanding fungal diversity in sustainable composite development.

The work aims to fabricate a sustainable mycelium-reinforced cellulose material by forming a DAC (hemp)–PVA hydrogel and cultivating *G. boninense*, *P. eryngii*, and *P. ostreatus* on its surface, chosen for their distinct mycelial architectures and reinforcing potential. The fabricated composites were systematically characterized for their structural, morphological, mechanical, thermal, and physico-chemical properties. By strategically leveraging their complementary strengths, this approach seeks to optimize the DAC–PVA composite design for improved performance, dimensional stability, and moisture resilience, thereby overcoming current MBC limitations and paving the way for applications in construction, biomedical devices, advanced packaging, and environmental sensing technologies.

## 2 Materials & methods

### 2.1 Used chemical reagents and preparation of materials

Hemp (*Cannabis sativa* L.) was provided as a gift from a local farm in Thailand. Sodium hydroxide (NaOH), sodium periodate (NaIO_4_), and hydrogen peroxide (H_2_O_2_) were procured from Kemaus Co., Ltd, Fisher Periodate Co., Ltd., and Chem-Supply Co., Ltd, respectively, for cellulose extraction and purification. Ethylene glycol (quenching agent) and hydrochloric acid (for pH adjustment) were obtained from QreC Co., Ltd. and Merck Co., Ltd., respectively. Polyvinyl alcohol (PVA; MW 85,000-124,000 g/mol), purchased from Chem-Supply Co., Ltd., served as the polymeric matrix. Phosphate buffered saline (PBS) and potato dextrose broth (PDB) were procured from Gibco, Co. Ltd. and Difco Co., Ltd., respectively. Hydroxylamine hydrochloride 99% (NH_2_OH∙HCl) was purchased from Loba Chemie Pvt. Ltd. All chemical reagents were used as received without further purification.

Three fungal species were used: *Ganoderma boninense*, *Pleurotus eryngii*, and *P. ostreatus*. *P. eryngii* and *P. ostreatus* mycelia were isolated from a mushroom cultivation bag and fresh mushrooms, respectively (local vendor). *G. boninense* was isolated from oil palm trees in Thailand. Pure cultures of each species were cultivated on potato dextrose agar (PDA; BD Difco™, USA) at 28 ± 2 °C for 7 days at the National Centre for Genetic Engineering and Biotechnology (BIOTEC-NSTDA). Cellulose extraction followed Sirichaibhinyo et al. [14] with minor modifications. Briefly, hemp fibers were nano-ground, then treated with 18% (w/v) NaOH at a 1 g:50 mL solid–liquid ratio, 80 °C for 2 h The slurry was subsequently oxidized with 5% (v/v) H_2_O_2_ at the same ratio and temperature/time, then bleached in 0.5 M NaOH + 5% (v/v) H_2_O_2_ (80 °C, 2 h). After each step, solids were recovered by suction filtration, thoroughly washed with distilled water, dried at 60 °C for 24 h, and stored in a desiccator.

**Fig. 1:**
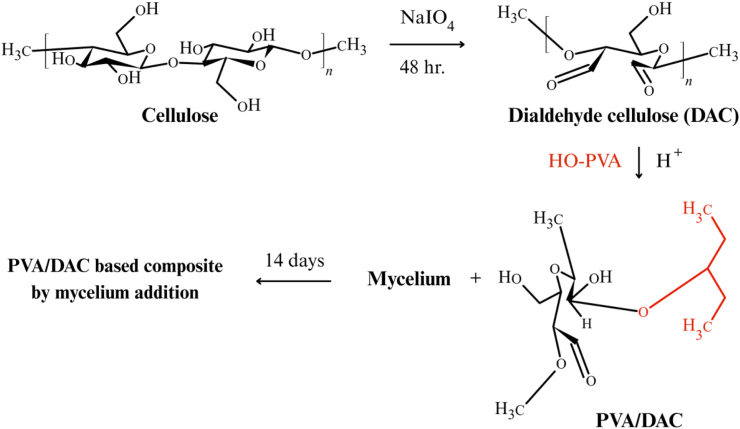
Schematic diagram and chemical reaction of cellulose from hemp and subsequent formation of mycelium-reinforced DAC-PVA composite.

Dialdehyde cellulose (DAC) was synthesized by oxidizing the purified cellulose. A 10 wt% NaIO_4_ solution was added to the cellulose suspension, pH adjusted to 3 (HCl) and stirred at ambient temperature (25 ± 2 °C) for 48 h in darkness. Ethylene glycol (2 mL) was added to quench unreacted periodate. DAC was recovered by centrifugation and dried (60 °C, 24 h). Fig. 1 illustrates the overall schematic for DAC preparation and subsequent composite formation. For PVA/DAC-mycelium composite fabrication, 10 g PVA was dissolved in 100 mL distilled water, and DAC was added (4% by weight relative to PVA). This 4% DAC loading was determined from preliminary trials to optimize mechanical strength and crosslinking without impairing flexibility or processability. HCl was added, and the mixture stirred (room temperature, 1 h), freeze-dried (48 h), then oven-dried (60 °C, 24 h). The dried composite material was cut into uniform pieces, soaked in PBS and PDB solutions (30 min each), and sterilized (autoclave, 121 °C, 15 min). Fifteen individual sterilized composite samples were prepared for each fungal species. Mycelial plugs (4-mm diameter, from 7-day old PDA cultures) were placed onto these samples (one plug per sample). Composites were incubated at 25 °C for 14 days to ensure robust mycelial growth and matrix reinforcement. From the fifteen colonized samples per fungal species, triplicate specimens were randomly selected for each characterization test.

### 2.2 Instruments and physico-chemical properties determination of mycelium-based composite (MBC)

^1^H NMR spectra were recorded in dimethyl sulfoxide (DMSO) using an Ascend™ 600/Avance III HD spectrometer (Bruker, Switzerland). FTIR spectra were acquired over the wavenumber range of 4000 to 500 cm^−^^1^ with a resolution of 2 cm^−^^1^ using an Invenio® FTIR spectrometer (Bruker) by using transmittance mode. The chemical state of the carbon (C) element was examined using an Axis Ultra DLD (Kratos, USA) with hybrid mode, operating at a step 100 meV, current 1.8 A and balance 2.6 V. The crystallinity was assessed using X-ray diffraction (XRD) with an AXS Model D8 Advance (Bruker, Germany), operating at a scan rate of 0.01°/step over a 2θ being 5 to 60°. Thermal decomposition (TGA) was performed by using a TGA55 thermogravimetric analyzer (TA Instruments, USA). The temperature was programmed to increase from 30 °C to 600 °C at a heating rate of 10 °C/min. Differential scanning calorimetry (DSC) was employed by using a DSC 3^+^ Differential scanning calorimeter (Mettler Toledo, Switzerland). The temperature was planned to increase from 25 °C to 600 °C at a heating rate of 10 °C/min. The morphological properties were examined using a scanning electron microscope (SEM; JEOL JSM7800F). SEM imaging was conducted at an accelerating voltage of 2 kV and a magnification of 2000X. Tensile strength was evaluated using a Universal Testing Machine (UTM) equipped with a 50 N load cell and a 50 mm probe, with a crosshead speed of 5 mm/min. Sample preparation was described in the supporting information. Wettability was assessed by measuring the contact angle using a Biolin Theta Lite Optical Tensiometers. The contact angle measurements were conducted to determine the surface properties of composite. The topological roughness surface was investigated using the NX10 atomic force microscope (Park Systems, South Korea).

The degree of oxidation (DO) was determined by reacting dialdehyde cellulose (DAC) with hydroxylamine hydrochloride (NH_2_OH·HCl), followed by titration to the initial pH with sodium hydroxide. In this procedure, 0.02 g of freeze-dried DAC was combined with 5 mL of 0.25 M hydroxylamine hydrochloride solution in deionized water, adjusted to pH 4.6. The mixture was agitated at room temperature for 48 h to facilitate the reaction of aldehyde groups with hydroxylamine. The hydrochloric acid released during the reaction was quantified by titration with sodium hydroxide. For analysis, 2 mL of the reaction mixture was diluted with 5 mL of deionized water, and the mixture was titrated with 0.1 M sodium hydroxide until the initial pH was restored. The blank value was determined by reacting 0.2 g of freeze-dried unmodified cellulose with 50 mL of 0.25 M hydroxylamine hydrochloride solution. The 8 mL solution of the resulting mixture was then titrated back to the initial pH. Each sample was prepared and analyzed four times to ensure measurement accuracy. The DO was calculated using the following equation (1).

(1) $${\text{DO}}_{\text{Titration}}\;\left(\%\right)=\left(V_{\text{NaOH}}\cdot \left[\text{NaOH}\right]\cdot M_{\text{AGU}}\cdot V_{1}/2\cdot m_{\text{DAC}}\cdot V_{0}\right)\times 100\%-{\text{DO}}_{\text{blank}}$$

In the equation, V_NaOH_ represents the volume of sodium hydroxide consumed during titration, [NaOH] is the concentration of the sodium hydroxide solution (0.1 M), and M_AGU_ corresponds to the molecular weight of the anhydroglucose unit (162 g/mol). The term m_DAC_ denotes the mass of the freeze-dried DAC sample, V_0_ is the initial volume of hydroxylamine hydrochloride solution added, and V_1_ is the volume of the reaction mixture used for titration. DO_blank_ refers to the degree of oxidation of the unmodified cellulose, which was used as a blank correction [45]. Additionally, the mechanism of this reaction was described as follows [46].

$$-\text{CHO}+{\text{NH}}_{2}\text{OH}\cdot \text{HCl}\;\to -\text{CHNOH}+\text{HCl}+H_{2}O$$

$$\text{HCl}+\text{NaOH}\;\to \;\text{NaCl}+H_{2}O$$

The gel fraction was determined by sectioning into four smaller pieces and air-dried in an oven at 60 °C for 24 h. The initial dry weight (W_i_) each sample was measured, followed by lyophilization until a constant weight (W_d_) was achieved. The gel fraction was calculated using the following equation (2):

(2) $$\text{Gel}\;\text{fraction}\;\left(\%\right)=\left(W_{d}\;/\;W_{i}\right)\times 100\%$$

The swelling behavior of the composites was assessed by determining the swelling ratio (SR). A dried sample was immersed in 50 mL of distilled water for 24 h. At predetermined time intervals, the swollen samples were withdrawn, the swelling ratio (SR) was calculated using the following equation (3):

(3) $$\text{Swelling}\;\text{ratio}\;\left(\%\right)=\left(W_{t}\;–\;W_{1}\right)/W1\;x\;100\%$$

where, W_1_ represents the weight of the dried sample and W_t_ denotes the weight of the swollen sample at various time intervals.

The equilibrium water content (EWC) was determined to evaluate their ability to absorb and retain water. A dried sample (W_s_) was immersed in 50 mL of distilled water and left for 24 h. At predetermined time intervals, the weight of the immersed sample (W_d_) was then measured. The EWC was calculated using the following equation (4):

(4) $$\text{EWC}\;\left(\%\right)=\left(W_{d}\;–\;W_{s}\right)/W_{s}\;x\;100\%$$

where W_s_ represents the weight of the dried sample and W_d_ denotes the weight of the immersed sample at various time intervals.

The biodegradability of the composites was assessed over a 28-day period. Pre-weighed, freeze-dried hydrogel samples (W_a_​) were individually suspended in sterile containers with 50 mL of PBS (pH = 7.4). These containers were incubated in a shaking water bath at 25 °C and 50 rpm. At specified time intervals (1, 4, 7, 14, 21, and 28 days), a set of triplicate samples for each composite type was retrieved from the PBS solution. The retrieved samples were thoroughly rinsed with distilled water to remove residual salts and then freeze-dried until a constant final dry weight (W_f_) was achieved. The degradation percentage at each time point was calculated using Equation (5): Degradation (%) = (W_a_ – W_f_)/W_a_ × 100% (5); Where W_a_ is the initial dry weight of the sample and W_f_ is its final dry weight after the respective incubation and freeze-drying period. All quantitative experiments were performed in triplicate, and data are presented as mean ± standard deviation (SD). Statistical significance of differences among mean values for mechanical properties (tensile strength, elongation at break), swelling ratio, equilibrium water content, gel fraction, and degradation rates were determined using one-way analysis of variance (ANOVA) followed by Tukey's HSD (Honestly Significant Difference) post-hoc test. Analyses were performed using SPSS version 30. A p-value <0.05 was considered statistically significant.

## 3 Results and discussion

### 3.1 Chemical analysis of dialdehyde cellulose from hemp

**Fig. 2:**
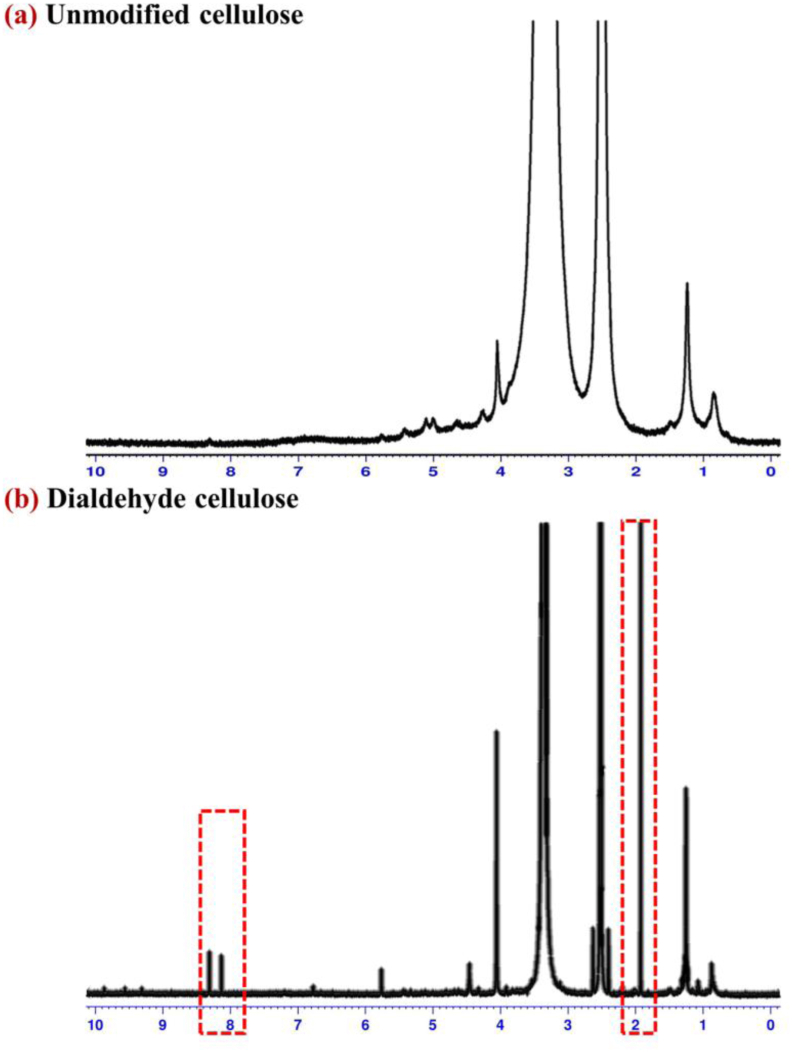
^1^H NMR spectra of dialdehyde cellulose (DAC) prepared from hemp.

Cellulose fiber was successfully extracted and purified from hemp, resulting in a fine, homogeneous powder with high crystallinity suitable for subsequent oxidation to dialdehyde cellulose (DAC) and yielding a fine, homogeneous powder. The extraction process effectively removed non-cellulosic components such as lignin and hemicellulose, as evidenced by the absence of colored residues and improved purity observed in the final product. The purified cellulose exhibited a high degree of crystallinity, which is essential for subsequent chemical modifications to produce DAC. This high crystallinity ensures enhanced structural integrity and reactivity, facilitating the incorporation of aldehyde functional groups necessary for the formation of robust MBCs. The ^1^H NMR spectrum of DAC (Fig. 2) exhibited a characteristic proton peak at approximately 1.900 ppm, corresponding to the protons at positions C2 or C3 of DAC [47], and an aldehyde proton resonance at around 9–10 ppm, consistent with previous observations by Simon et al. (2022) [45]. This slight shift in aldehyde proton resonance is likely due to structural transformations of DAC into aldehyde hydrate forms (-CH(OH)_2_) when dissolved in an aqueous solution, as reported by Lázaro Martínez et al. (2010) [48].

Fig. 2 indicates the ^1^H NMR spectra of purified cellulose and DAC derived from hemp. It can be used to confirm the chemical structure of DAC. It was evident that DAC was successfully prepared. The ^1^H NMR of DAC present in Fig. 2 shows a peak at 1.900 ppm associated with the proton found on the DAC's C2 or C3 [47] and represents a peak around 8 ppm, which referred to the proton of aldehyde. However, the peak was slightly shifted down to the range of 9–10 ppm. This was probably because DAC can transform its structure to aldehyde forms (-CH(OH)_2_) when dissolve in aqueous solution while testing [45,48].

**Fig. 3:**
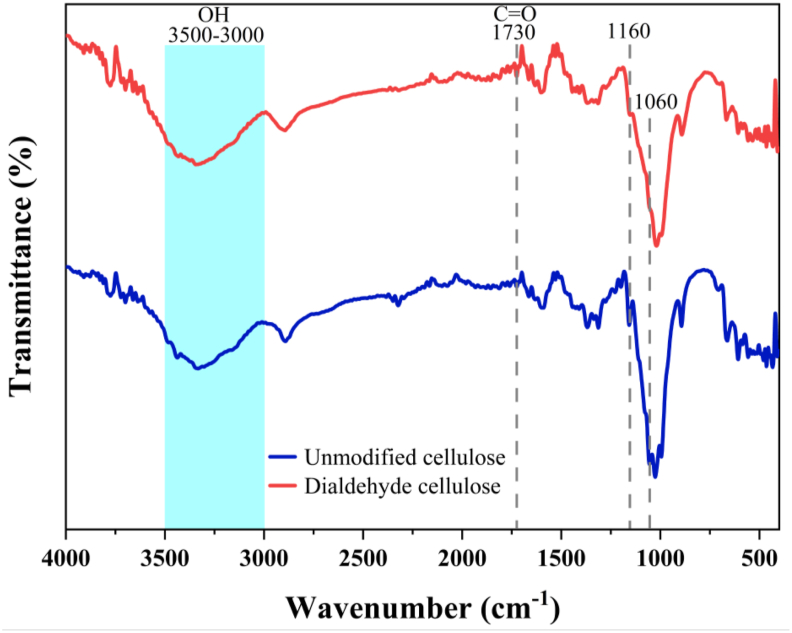
FTIR spectra of dialdehyde cellulose (DAC) prepared from hemp.

The FTIR spectra (Fig. 3) further confirmed successful DAC synthesis from cellulose, showing characteristic peaks at 1060 and 1130 cm^−1^, indicating reduced hydroxyl (O–H) groups, consistent with findings by Chen et al. (2023). The distinct peak at approximately 1730 cm^−1^, attributed to the aldehyde (C=O) functional group, strongly validates successful cellulose oxidation, aligning with the observations of Lee et al. (2020) [49]. These results indicate that aldehyde groups were effectively introduced onto the cellulose backbone, facilitating enhanced hydrogen bonding and improved compatibility with PVA in composite formulations. Collectively, these comparative analyses reinforce the robustness and reproducibility of the chemical modification procedures employed. Furthermore, the degree of oxidation of DAC and unmodified cellulose were 3.954 ± 0.42 and 0.096 ± 0.01, respectively. An increase in the degree of oxidation resulted in a higher number of reactive aldehyde groups in DAC, thereby increasing the potentiality of covalent bond formation with polyvinyl alcohol (PVA) chains. The crosslinking mechanism typically involved the formation of acetal or hemiacetal linkages between the aldehyde groups of DAC and the hydroxyl groups of PVA under suitable conditions. This chemical crosslinking produced a three-dimensional polymer network, which enhanced mechanical strength, thermal stability, and water resistance compared to physically blended systems. In contrast, unmodified cellulose primarily interacted with PVA through hydrogen bonding, resulting in weaker intermolecular interactions and reduced network stability [[50], [51], [52]].

**Fig. 4:**
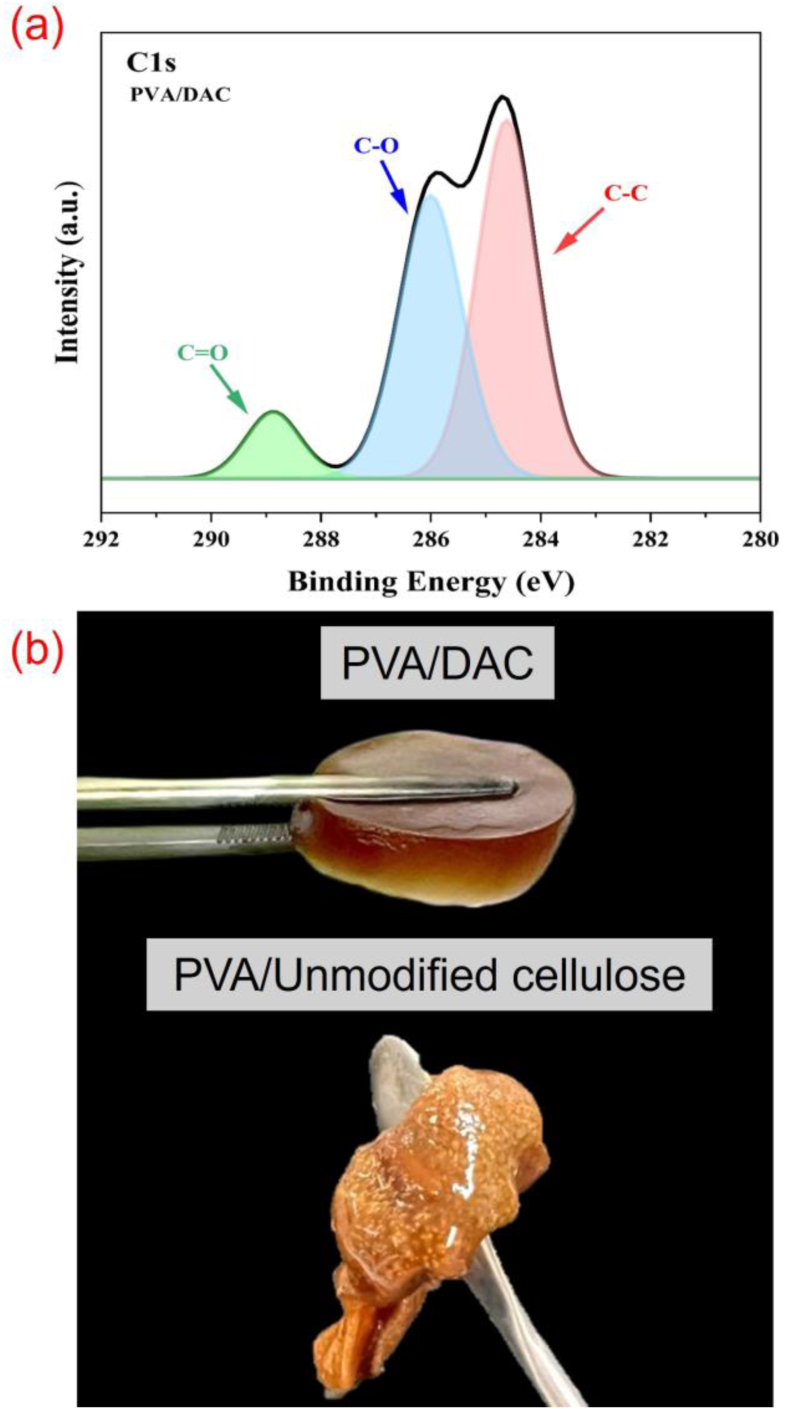
(a) XPS spectra of dialdehyde cellulose-polyvinyl alcohol (DAC-PVA) composite and (b) The composites after autoclaving at 121 °C for 15 min.

Fig. 4a indicates XPS spectra of PVA/DAC composite; the C1s spectra displayed two prominent peaks corresponding to the C–C and C–O bonds. In contrast, a minor peak from the aldehyde group of DAC suggested that the dialdehyde group interacts with the hydroxyl group to create an acetal bond under the influence of HCl. Establishing an acetal bond leaded to a considerable enhancement in the intensity of the C–O bond peak in PVA/DAC [53]. In addition, Fig. 4b shows the composite materials after autoclaving at 121 °C for 15 min, the PVA/DAC composite retained its shape, whereas the PVA-Unmodified cellulose composite melted. This phenomenon revealed the strong crosslinked structure of DAC and PVA.

### 3.2 Properties determination of mycelium-based composite

Hydrogel composites incorporating PVA, DAC, and mycelia from *Pleurotus eryngii* (PVA/DAC/E), *Ganoderma boninense* (PVA/DAC/L), and *Pleurotus ostreatus* (PVA/DAC/O) were successfully fabricated. All fabricated hydrogels, including those with mycelial reinforcement, exhibited excellent elasticity upon initial handling, capable of being stretched and reforming to their original shape. This indicated robust intermolecular interactions, likely enhanced by the presence of DAC and the mycelial networks The general physical characteristics aligned with previous reports [49]. While subsequent analyses revealed species-specific impacts, initial observations suggested successful composite formation across all mycelial types. While initial visual assessments and broad spectroscopic features (FTIR, XRD) suggested successful composite formation with common polymeric backbones across all mycelial types, subsequent detailed thermal, mechanical, and physico-chemical analyses (discussed below) revealed distinct species-specific impacts on the composite properties.

**Fig. 5:**
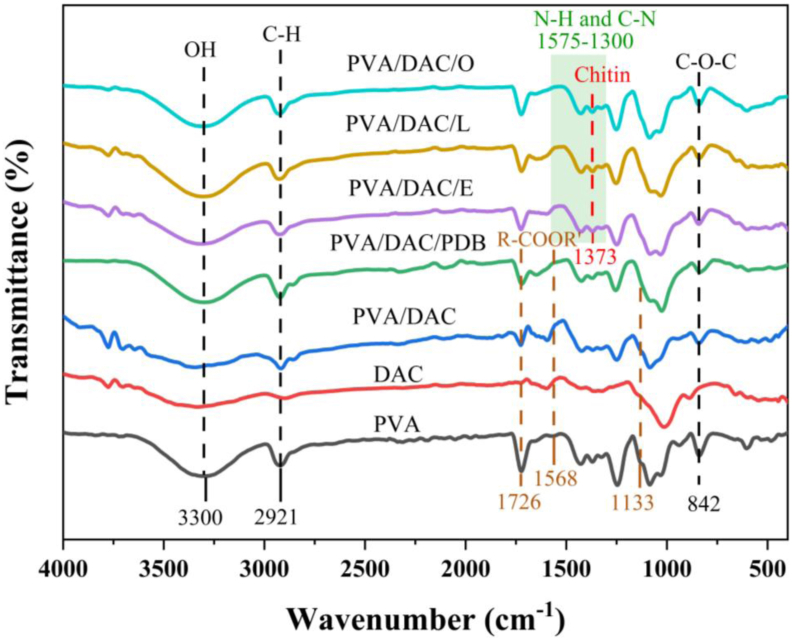
FTIR spectra of polyvinyl alcohol (PVA), dialdehyde cellulose (DAC), dialdehyde cellulose-polyvinyl alcohol (DAC-PVA) and mycelium-based composite (MBC) and label name at PVA, DAC, PVA/DAC, PVA/DAC/PDB (potato dextrose broth), PVA/DAC/E (*Pleurotus eryngii*), PVA/DAC/L (*Ganoderma boninense*), and PVA/DAC/O (*Pleurotus ostreatus*).

Fig. 5 presents FTIR spectra of DAC–PVA and MBC samples. All characteristic curves exhibited similar features, indicating the presence of common functional groups across the composites. Notably, peaks were observed at wavenumbers of 3300 cm^−1^ and 2921 cm^−1^, which are attributed to O–H stretching and C–H stretching vibrations, respectively. The presence of hydroxyl groups suggests that the hydrogel composites can effectively adhere to water molecules through hydrogen bonding. Subsequent peaks at 1726 cm^−1^ and 1568 cm^−1^ were identified, likely due to the presence of C–O stretching vibrations. These peaks correspond to the carbonyl groups located on the side chains of DAC, confirming successful oxidation of cellulose. Additionally, peaks observed at 1133 cm^−1^ and 842 cm^−1^ correspond to C–O–C stretching vibrations. The peak at 842 cm^−1^ is typically associated with the presence of hemicellulose, which is the most easily degraded component of fungal cell walls, as suggested by Liu et al. (2019) [54]. These characteristic peaks align with previous findings reported by Chen et al. (2023), further validating the structural integrity of the composites [55]. Furthermore, the integration of three mycelial types—*Ganoderma boninense*, *Pleurotus eryngii* and *Pleurotus ostreatus*—into the hydrogel composite resulted in the emergence of a distinct absorption peak at 1373 cm^−1^. This peak is indicative of N–H stretching vibrations, which is likely attributable to the presence of chitin within the mycelial structure [33,56].

The incorporation of chitin not only enhances the mechanical properties of the composite but also contributes to its biocompatibility and structural stability. Overall, the FTIR analysis confirms the successful formation of DAC and its effective integration into the PVA matrix, as well as the incorporation of mycelial components. The presence of aldehyde and chitin functional groups plays a crucial role in enhancing the composite's mechanical strength and water retention capabilities, making them suitable for various structural and functional applications.

**Fig. 6:**
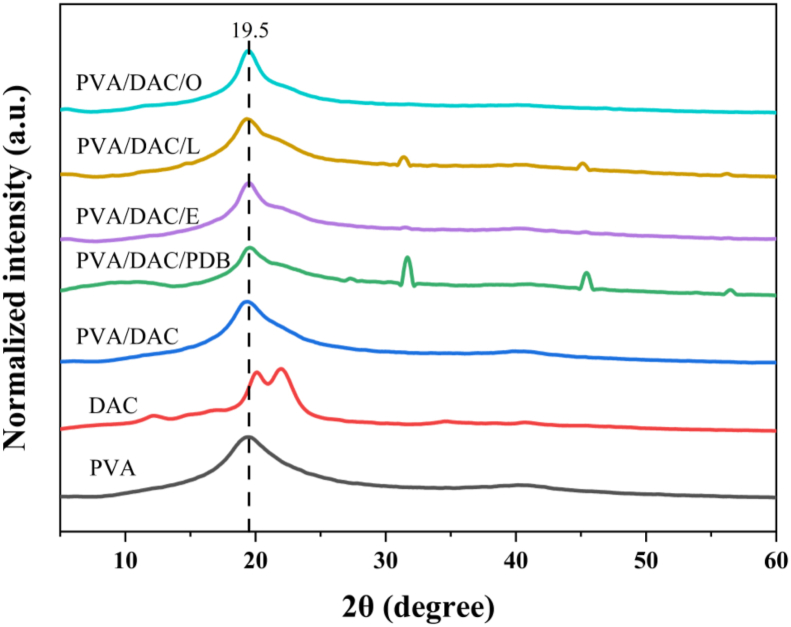
XRD spectra of polyvinyl alcohol (PVA), dialdehyde cellulose (DAC), dialdehyde cellulose-polyvinyl alcohol (DAC-PVA) and mycelium-based composite (MBC) and label name at PVA, DAC, PVA/DAC, PVA/DAC/PDB (potato dextrose broth), PVA/DAC/E (*Pleurotus eryngii*), PVA/DAC/L (*Ganoderma boninense*), and PVA/DAC/O (*Pleurotus ostreatus*).

XRD patterns (Fig. 6) for PVA, DAC, PVA/DAC, and MBCs revealed that all PVA-containing samples exhibited a prominent broad peak at 2θ = 19.5°, characteristic of PVA's semi-crystalline structure [57]. The incorporation of DAC and mycelium did not significantly alter this primary PVA peak, suggesting the mycelium was well-integrated within the matrix without forming large, distinct crystalline domains or substantially changing the bulk PVA crystallinity, likely due to the relatively small amount of mycelium [58].

**Fig. 7:**
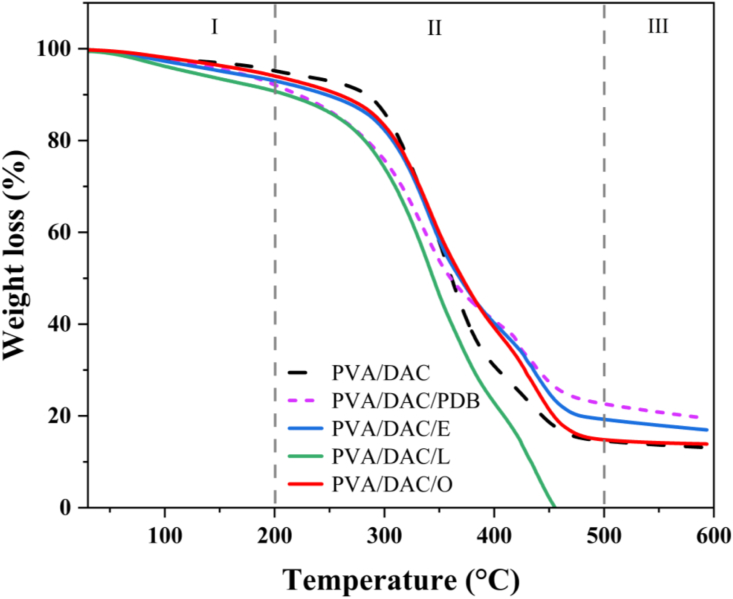
Thermal decomposition (TGA) behavior of dialdehyde cellulose-polyvinyl alcohol (DAC-PVA) and mycelium-based composite (MBC) and label name at PVA/DAC, PVA/DAC/PDB (potato dextrose broth), PVA/DAC/E (*Pleurotus eryngii*), PVA/DAC/L (*Ganoderma boninense*), and PVA/DAC/O (*Pleurotus ostreatus*).

Fig. 7 illustrates the thermal decomposition behavior of DAC-PVA and MBCs. This analysis enabled the assessment of the thermal resistance properties of the samples when exposed to elevated temperatures. For comparative purposes, pristine DAC and PVA-based hydrogel composites were also included. All samples exhibited similar thermal decomposition profiles, characterized by three distinct regions upon application of external heat. In the temperature range from room temperature to 200 °C, approximately 10 wt% loss was observed, likely attributable to the evaporation of water. This indicates that hydrogel composites have an inherent affinity for water molecules, facilitating hydrogen bond interactions. To avoid degradation, and bacterial damage, it is recommended that the hydrogels be stored at low temperatures, consistent with the findings of Cesar et al. (2023) [59].

Between 200 °C and 500 °C, a broad region of weight loss was observed, corresponding to the pyrolysis of organic structures within the composites. The hydrogel composites, primarily composed of organic components, undergo thermal degradation, resulting in the formation of volatile gases such as CO_2_ and NO_x_. At temperatures above 500 °C, only about 10 wt% loss was observed, attributed to the formation of char and residual materials, as reported by Nigmatullin et al. (2004) [60]. These results indicate that the composites possess considerable thermal stability, making them suitable for applications requiring resistance to high temperatures.

**Fig. 8:**
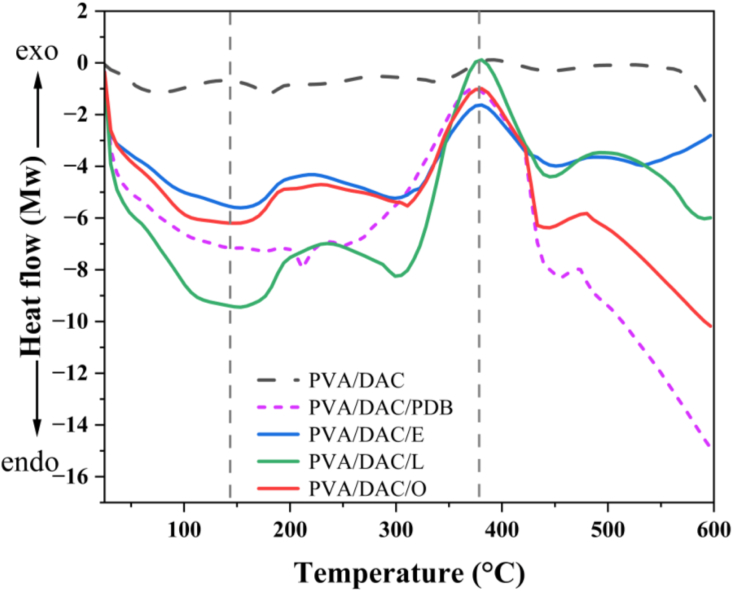
Differential scanning calorimetry (DSC) thermogram during heating of dialdehyde cellulose-polyvinyl alcohol (DAC-PVA) and mycelium-based composite (MBC) and label name at PVA/DAC, PVA/DAC/PDB (potato dextrose broth), PVA/DAC/E (*Pleurotus eryngii*), PVA/DAC/L (*Ganoderma boninense*), and PVA/DAC/O (*Pleurotus ostreatus*).

Fig. 8 illustrates the thermal properties, including melting (T_m_) and crystallization (T_c_) behaviors, of various composite materials. A sharp endothermic melting peak is observed for the DAC and PVA-based composites with added mycelium, occurring at approximately 140 °C, with enthalpy changes (ΔH) of around 124 J/g for *Pleurotus eryngii*, 118 J/g for *Ganoderma boninense*, and 143 J/g for *Pleurotus ostreatus*. These results show minor variations between the mycelium species. Furthermore, all mycelium-enhanced composites exhibit pronounced exothermic crystallization peaks at approximately 380 °C, with enthalpy changes of around 171 J/g, 172 J/g, and 256 J/g, respectively. This behavior is attributed to the specific mycelium species and the pre-treatment of the composite material in potato dextrose broth (PDB), a culture medium used prior to mycelium inoculation. In contrast, the original composite material (PVA/DAC) shows two distinct endothermic melting peaks at 73.96 °C and 180.27 °C, with enthalpy changes of 87.70 J/g and 30.63 J/g, respectively, indicating weaker mechanical properties compared to the mycelium-enhanced composites.

**Fig. 9:**
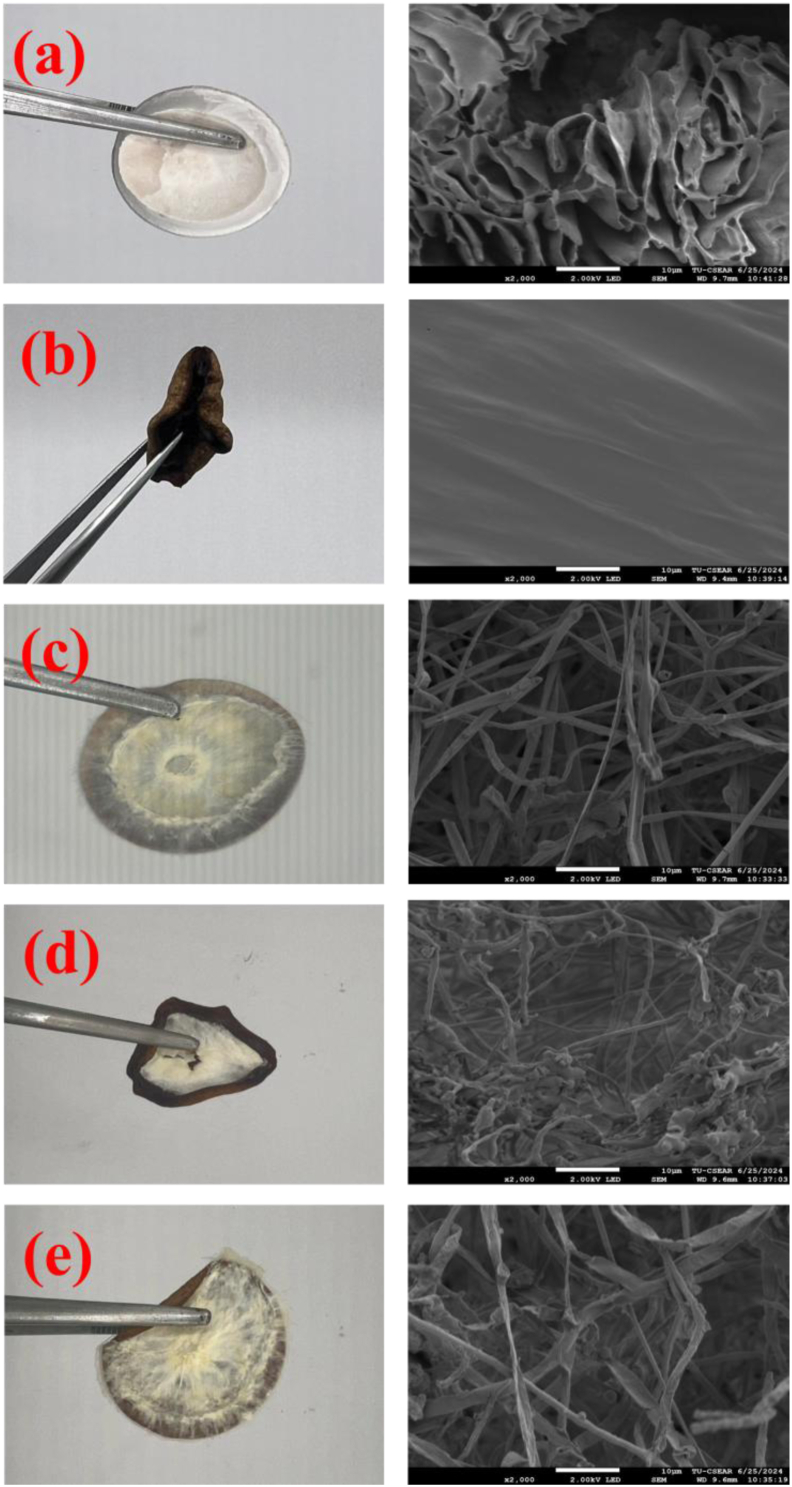
(Left) External characteristic (right); morphological properties of dialdehyde cellulose-polyvinyl alcohol (DAC-PVA) and MBCs; (a) PVA/DAC, (b) PVA/DAC/PDB (potato dextrose broth), (c) PVA/DAC/E (*Pleurotus eryngii*), (d) PVA/DAC/L (*Ganoderma boninense*), and (e) PVA/DAC/O (*Pleurotus ostreatus*).

In addition, Fig. 9 (left) presents the bendable characteristics of DAC-PVA and MBCs. All samples containing the three types of mycelia—*Ganoderma boninense*, *Pleurotus eryngii*, and *Pleurotus ostreatus*—demonstrated excellent bendable behavior under applied external loads. This flexibility suggests that these composites have potential for various applications, aligning with the previous works of Antinori et al. (2020, 2021) [61,62]. Correspondingly, scanning electron microscopy (SEM) revealed the morphological properties (Fig. 9, right panels). The PVA/DAC control (Fig. 9a, right panel) showed a relatively dense structure, while the mycelium-based composites (Fig. 9c–e, right panels) clearly displayed an interwoven network of fungal hyphae integrated within the polymer matrix, confirming effective mycelial colonization. The microstructure of the composites revealed an oriented arrangement of DAC fibers, which were randomly distributed within the composite network. The interface between fibers were introduced due to the repulsive forces of hydroxyl groups, acting as pendent groups alongside the cellulose chains. This morphological configuration is consistent with previous studies by Nigmatullin et al. (2004), confirming the effective integration of DAC and mycelial networks within the PVA matrix [60]. The random distribution and interfacial bonding contribute to the composite's mechanical strength and structural integrity, enhancing its suitability for practical application.

**Fig. 10:**
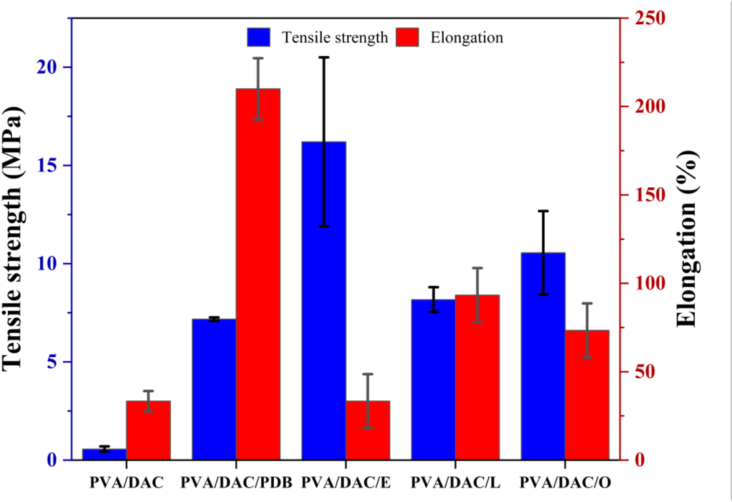
Mechanical properties of dialdehyde cellulose-polyvinyl alcohol (DAC-PVA) and MBC; and label name at PVA/DAC, PVA/DAC/PDB (potato dextrose broth), PVA/DAC/E (*Pleurotus eryngii*), PVA/DAC/L (*Ganoderma boninense*), and PVA/DAC/O (*Pleurotus ostreatus*).

The mechanical properties of DAC-PVA and MBCs were evaluated using a Universal Testing Machine (UTM), as reported in Fig. 10. This instrument facilitated the determination of tensile strength and elongation at break of the composite. It was observed that the incorporation of mycelium into the DAC-PVA matrix significantly enhanced both tensile strength and elongation at break. The mycelium acts as a reinforcing agent, forming strong bonds at the interface between DAC and polyvinyl alcohol, thereby improving the composite's mechanical integrity. Specifically, the composite containing *Pleurotus eryngii*, exhibited the highest tensile strength, while the composite with *Ganoderma boninense* demonstrated the maximum elongation at break. These results indicate that different mycelial species contribute uniquely to the mechanical properties of the composites, likely due to variations in their structural networks and bonding capabilities. The enhanced tensile strength suggests improved load-bearing capacity, whereas increased elongation at break indicates greater flexibility and ductility of the composites. These findings are consistent with the results reported Appels et al. (2018), who demonstrated the beneficial effects of mycelial integration on the mechanical performance of composite materials. The observed differences in mechanical performance among the MBCs can be attributed to the distinct morphological characteristics of the fungal species [34]. *Pleurotus eryngii* demonstrated the highest tensile strength due to its thick, densely packed hyphae, which form a rigid and well-aligned network within the DAC–PVA matrix. This structural organization enhances interfacial adhesion and facilitates efficient stress transfer, thereby increasing the load-bearing capacity of the composite. In contrast, *Ganoderma boninense* exhibited the greatest elongation at break, likely due to its more flexible, branched hyphal architecture and the production of extracellular polysaccharides (EPS). These features contribute to a more extensible composite structure that allows greater deformation under stress before failure. Thus, while *P. eryngii* reinforces the composite through stiffness and strength, *G. boninense* enhances ductility and flexibility, demonstrating the species-specific tuning potential of fungal mycelia in bio-based composite design [34,35].

**Fig. 11:**
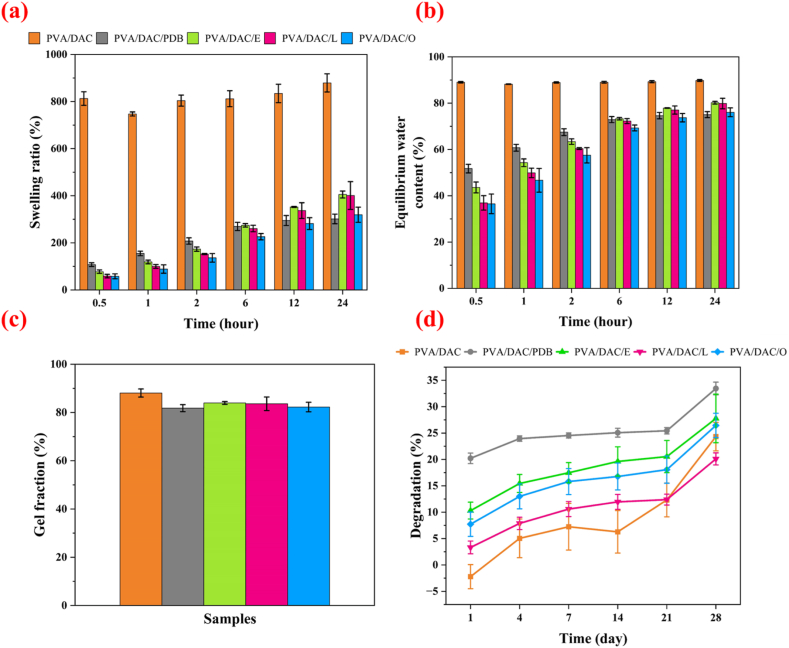
(a) Swelling behavior (b) equilibrium water content (c) gel fraction and (d) degradation rate of dialdehyde cellulose-polyvinyl alcohol (DAC-PVA) and MBC; and label name at PVA/DAC, PVA/DAC/PDB (potato dextrose broth), PVA/DAC/E (*Pleurotus eryngii*), PVA/DAC/L (*Ganoderma boninense*), and PVA/DAC/O (*Pleurotus ostreatus*).

Fig. 11 exhibits the physico-chemical properties of DAC-PVA and MBCs, focusing on water-swelling behavior, equilibrium water content (EWC), and gel fraction. The evaluations were conducted over a 24-h period to comprehensively assess the composites' interactions with water. In the swelling behavior assessment, the pristine DAC-PVA composite exhibited a high swelling ratio of approximately 800%, indicating a substantial capacity to absorb water. In contrast, all MBCs demonstrated significantly lower swelling ratios, ranging between 200% and 300%. This reduction in swelling capacity can be attributed to the incorporation of mycelium, which provides additional dimensional stability and restricts excessive water uptake. Over the investigation period, a slight increase in the swelling ratio was observed in MBCs, likely due to gradual infiltration of water molecules into the composite network. This behavior underscores the role of mycelium in modulating the hydrogel's swelling properties, enhancing its suitability for applications where controlled water absorption is essential. Consistent with the swelling behavior results, the equilibrium water content (EWC) of the composites showed a similar trend. The pristine DAC-PVA composite maintained a high EWC of 90%, reflecting its high-water retention capability. Conversely, MBCs exhibited a reduced EWC ranging from 40% to 60%. The decrease in EWC with mycelium incorporation suggests enhanced structural integrity and reduced free water availability within the composite network. This reduction is beneficial for applications requiring stable water content and minimized swelling, thereby preventing potential degradation or mechanical instability. The gel fraction analysis revealed that all samples, including both pristine DAC-PVA and MBCs, maintained a high gel fraction between 80% and 90 wt%.

This high gel fraction indicates effective crosslinking within the hydrogel matrix, ensuring that only 10 wt% of water remains within the composite network. The presence of mycelium did not adversely affect the gel fraction, suggesting that the integration of mycelial networks does not compromise the crosslinking efficiency of the hydrogel. Instead, it likely contributes to a more robust and interconnected network, enhancing the composite's mechanical and structural properties. The incorporation of mycelium plays a crucial role in enhancing the composite properties by forming chemical bonds between DAC and PVA throughout the hydrogel network [26]. The mycelial networks act as reinforcing agents, providing additional crosslinking points that improve dimensional stability and mechanical strength. This synergistic interaction not only reduces excessive swelling and water content but also maintains a high gel fraction, ensuring the composite's structural integrity and durability. These findings are consistent with previous studies reported by Appels et al. (2018), which demonstrated that the integration of mycelial networks into polymeric matrices significantly enhances mechanical and physico-chemical properties [34]. The observed trends in swelling behavior, EWC, and gel fraction align with the established benefits of mycelial reinforcement in composite materials, validating the effectiveness of mycelium as a functional additive in hydrogel-based composites.

In addition, the MBCs were degraded within 3 to 4 weeks with a 10–25% degradation rate. The rate was superior to pristine PVA/DAC because mycelium was considered as a naturally occurring organic matter that microorganisms can effortlessly consume and break down. Conversely, PVA/DAC is also biodegradable. It requires specific environmental conditions alongside some microorganism actions to degrade. The biodegradability of the composites in PBS was monitored over 28 days (Fig. 11d). The pristine PVA/DAC composite exhibited minimal degradation, with approximately 5% weight loss by day 28. In contrast, all mycelium-based composites (MBCs) demonstrated significantly higher degradation. Specifically, by day 28, composites reinforced with *P. eryngii* (PVA/DAC/E), *G. boninense* (PVA/DAC/L), and *P. ostreatus* (PVA/DAC/O) showed weight losses in the range of 20–35%, with distinct degradation profiles for each species. The PVA/DAC/PDB control also showed considerable degradation, greater than PVA/DAC alone. These findings suggest that mycelial incorporation enhances the susceptibility of the composites to breakdown in PBS, indicating improved biodegradability compared to the PVA/DAC matrix alone.

**Fig. 12:**
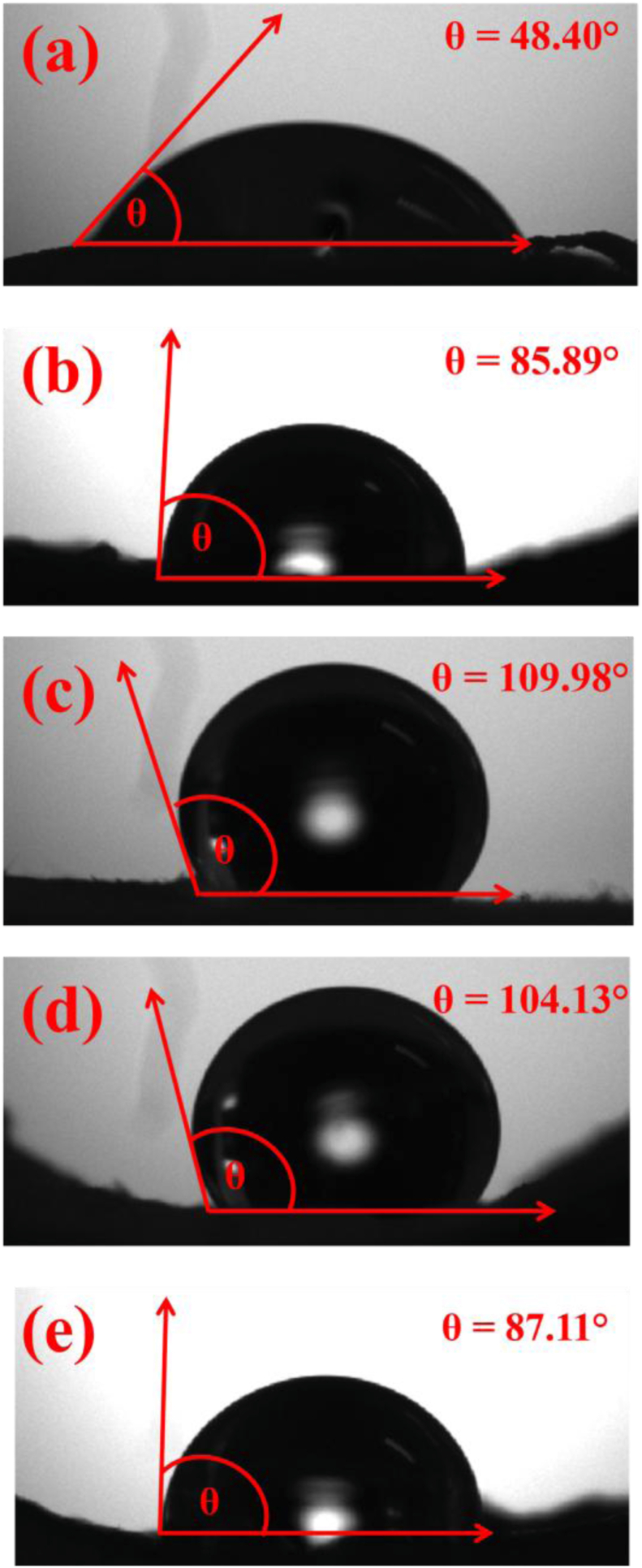
Contact angle measurement of dialdehyde cellulose-polyvinyl alcohol (DAC-PVA) and mycelium-based composite (MBC); (a) PVA/DAC, (b) PVA/DAC/PDB (potato dextrose broth), (c) PVA/DAC/E (*Pleurotus eryngii*), (d) PVA/DAC/L (*Ganoderma boninense*), and (e) PVA/DAC/O (*Pleurotus ostreatus*).

The incorporation of mycelium into the DAC–PVA composites significantly increased the water contact angle, shifting surface characteristics from hydrophilic to distinctly hydrophobic (Fig. 12). This transformation is primarily due to the hydrophobic nature of fungal mycelia, particularly resulting from the lipid-rich components and chitinous structures of fungal cell walls. Hydrophobic surface modification enhances moisture resistance, minimizes water uptake, and thereby substantially improves dimensional stability and long-term durability. Such surface properties are highly relevant in industrial applications where water-resistance is crucial, including sustainable packaging materials, biodegradable construction components, and outdoor environmental sensors. Environmentally, this reduced moisture sensitivity also extends the lifetime of materials in humid conditions, lowering replacement rates and waste generation, thus supporting sustainable material utilization and circular economy practices.

In the case of composites without mycelium, the contact angle was measured to be 48.40°, indicating hydrophilic characteristics. This low contact angle suggests that the pristine DAC-PVA composites readily adhere to water molecules through hydrogen bonding, facilitating excellent water uptake and interaction. Conversely, the incorporation of mycelium into the composite matrix resulted in a significant increase in the contact angle, ranging from 85° to 110°. This shift towards higher contact angles indicates a transition from hydrophilic to hydrophobic behavior, imparting the composites with water-resistant properties. This transformation is attributed to the mycelium's cell membrane, which features a lipid bilayer known for its hydrophobic characteristics. The enhanced wettability and surface modulation observed at the membrane surface and the mycelium-membrane interface indicate improved hydrophilicity. Notably, the membrane-mycelium interface exhibited a hydrophilic character, while the opposing hydrophobic side was characterized by a densely packed layer of fibrous hyphae [30,44]. The enhanced hydrophobicity is attributed to the presence of mycelial networks, which are likely to introduce hydrophobic functional groups and disrupt the hydrogen bonding network within the hydrogel matrix.

This modification in surface properties not only reduces water absorption but also enhances the durability and stability of the composites in aqueous environments. The observed increase in contact angle upon mycelium incorporation aligns with previous studies by Antinori et al. [61,62], who demonstrated that mycelial integration can effectively modulate the surface wettability of polymeric composites, thereby broadening their application potential in areas requiring moisture resistance and structural integrity. Overall, the contact angle measurements confirm that mycelium incorporation plays a pivotal role in tailoring the surface properties of DAC–PVA composites, enhancing their suitability for diverse applications ranging from biomedical devices to environmental sensors. Furthermore, the transition from hydrophilic to hydrophobic behavior following mycelium incorporation into the DAC–PVA matrix is attributed to both biochemical alterations and microstructural transformations within the composite. Fungal mycelia inherently contain chitin, hydrophobic proteins, and lipids—components with low surface energy that naturally repel water [33,62,63]. When introduced into the DAC–PVA network, these hydrophobic biomolecules partially mask or replace the polymer's hydrophilic hydroxyl and aldehyde groups, thereby reducing hydrogen bonding capacity with water molecules. Simultaneously, the formation of an entangled hyphal network contributes to increased surface roughness and porosity at both the micro and nanoscale levels [64]. This textured surface promotes the entrapment of air pockets between the water droplets and the material, giving rise to the Cassie–Baxter state, in which the liquid rests on a composite interface of solid and air, effectively minimizing the solid–liquid contact area and increasing the water contact angle [65].

Additionally, during active mycelial growth, fungi excrete extracellular polymeric substances (EPS) comprising glycoproteins, polysaccharides, and fatty acids that further lower the surface energy and introduce a hydrophobic barrier. These EPS layers protect fungal colonies and coat the surrounding material, leading to enhanced surface water resistance. Importantly, the hydrophobicity is not solely a result of surface chemistry but also a function of the hierarchical structure and orientation of the fungal hyphae, which create physical barriers to water penetration. These combined effects—chemical masking of hydrophilic groups, structural roughness, EPS deposition, and the Cassie–Baxter effect—synergistically transform the initially hydrophilic DAC–PVA surface into one that exhibits robust hydrophobicity. This shift plays a critical role in improving the composite's dimensional stability, moisture resistance, and long-term durability under humid or wet conditions.

**Fig. 13:**
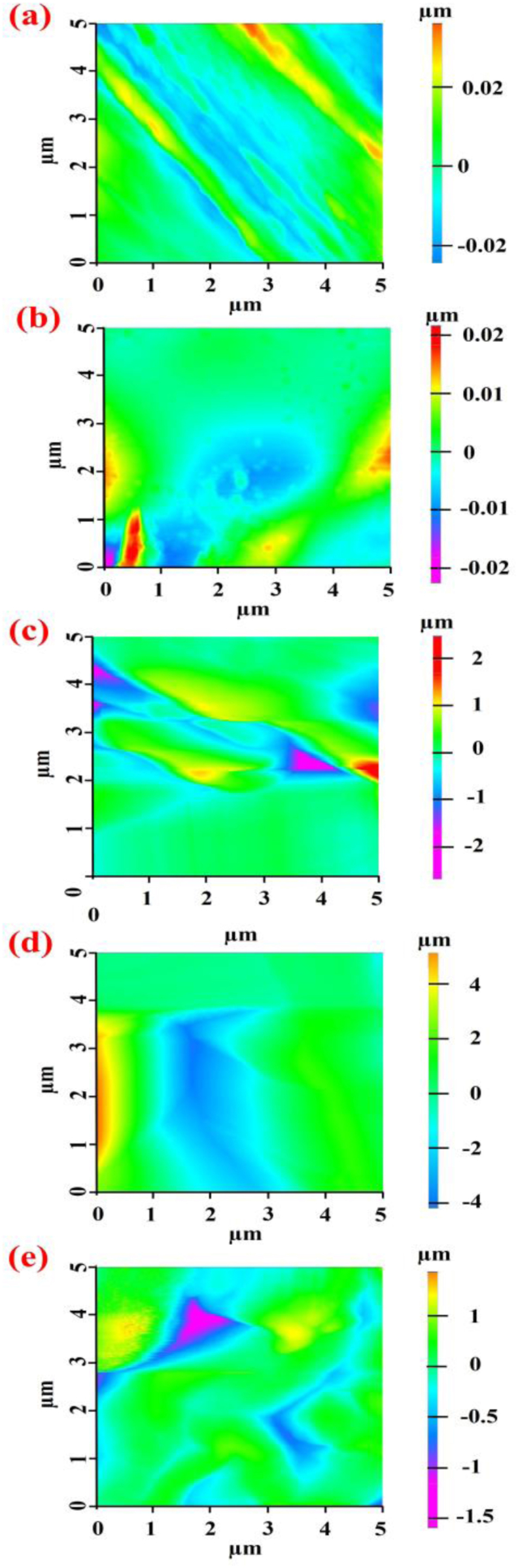
AFM image of dialdehyde cellulose-polyvinyl alcohol (DAC-PVA) and mycelium-based composite (MBC); (a) PVA/DAC, (b) PVA/DAC/PDB (potato dextrose broth), (c) PVA/DAC/E (*Pleurotus eryngii*), (d) PVA/DAC/L (*Ganoderma boninense*), and (e) PVA/DAC/O (*Pleurotus ostreatus*).

Fig. 13 indicates the surface roughness of DAC-PVA and MBCs; the presence of three types of mycelia—*Ganoderma boninense*, *Pleurotus eryngii*, and *Pleurotus ostreatus*—demonstrated covering the PVA-DAC surface; hence, the surface roughness increased significantly due to the clear difference in height variation and surface structural irregularities compared to PVA/DAC and PVA/DAC/PDB. It exhibited peaks and valleys distributed in multiple directions, indicating a higher roughness amplitude. The amplitude range of MBCs was −4 to 4 μm, whereas that of PVA/DAC and PVA/DAC/PDB was −0.2 to 0.2 μm. The increase in surface roughness observed in the MBCs composites can be interpreted through the Cassie–Baxter theory. According to this model, the presence of hierarchical micro- and nano-structures on a surface can effectively trap air within surface grooves, especially when combined with low surface energy. This entrapment of air pockets significantly reduced the effective solid-liquid contact area, thereby elevating the water contact angle and diminishing the material's overall wettability [30,66]. Consequently, the more complex and irregular surface topography introduced by the mycelium was more conducive to establishing a Cassie–Baxter state compared to the PVA/DAC system. This structural modification promoted a synergistic effect that enhanced the composite's hydrophobicity and water resistance, provided that the surface was concurrently characterized by chemical functional groups with low surface energy.

We further interpret the observed increases in apparent hydrophobicity by considering hydrophobins—amphipathic fungal proteins that self-assemble at interfaces and are known to render hyphae and colonized surfaces water-repellent—alongside previously discussed contributors such as chitin, lipids/extracellular polymeric substances (EPS), and Cassie–Baxter surface-roughness effects. In our system, species-dependent deposition of hydrophobins on (or within) the DAC–PVA surface could synergize with topographical changes from mycelial growth to elevate contact angles and reduce wetting. To disentangle these mechanisms, future work will quantify hydrophobin signatures and their functional impact via (i) partitioning SDS-soluble vs. SDS-insoluble protein fractions and (iii) contact-angle hysteresis measurements before/after protease treatment, thereby directly testing the contribution of proteinaceous surface assemblies relative to polysaccharide/lipid components and roughness. We note the darkened/black coloration of the composites after growth-stop and drying, which likely reflects a combination of medium residues (e.g., PDB components concentrated or thermally modified during processing) and fungal pigments/metabolites deposited at the surface. The relevance of color is application-dependent: for biomedical/food-adjacent uses, we recommend leachables profiling (UV–Vis of rinsates; targeted LC–MS for pigment/metabolite signatures) together with cytocompatibility screening; for packaging/industrial uses, color is typically secondary to moisture/mechanical performance, though consistency can be improved through strain and medium control and optional post-treatments (e.g., mild bleaching or surface sealing) to stabilize appearance under handling and moisture-cycling conditions.

In terms of scalability and commercialization, the DAC–PVA platform is compatible with established casting/curing workflows (batch trays or roll-to-roll lines) and freeze-drying where porosity is desired, with mycelial growth staged post-cure in controlled humidity/temperature chambers. The matrix tolerates steam/thermal sterilization cycles commonly used in bioprocessing, and growth-stop plus drying can be integrated into continuous ovens. Quality assurance can leverage routine monitoring of the degree of oxidation (DAC), gel fraction, residual aldehyde content, moisture uptake, and mechanical benchmarks to ensure batch-to-batch consistency. Cost and sustainability considerations include the price and availability of cellulose feedstock, periodate use and recovery/regeneration, and PVA grade, with periodate recycling and water reuse reducing the environmental footprint. Near-term applications include water-resistant flexible films, biodegradable packaging substrates, and sensor/biomaterial supports where moderate strength, tunable hydrophobicity, and bio-integration are advantageous. Key risks—such as variability in fungal morphology and pigmentation—can be mitigated via strain selection, tight control of growth parameters, and optional surface sealing to enhance colorfastness and stability under moisture cycling.

## 4 Conclusions

This study successfully developed mycelium-reinforced cellulose composites by integrating hemp-derived DAC with PVA and fungal mycelia from *Ganoderma boninense*, *Pleurotus eryngii*, and *P. ostreatus*. The resulting composites exhibited significant improvements in mechanical properties, with *P. eryngii* enhancing tensile strength and *G. boninense* notably increasing flexibility. Mycelial incorporation also led to enhanced thermal stability, significantly reduced moisture sensitivity due to a shift to hydrophobic surface characteristics and improved dimensional stability. These attributes make them highly suitable for applications in sustainable packaging, biodegradable construction materials, biomedical scaffolds, and environmental sensors, particularly where mechanical durability, thermal resilience, and moisture resistance are critical. Future research should focus on optimizing fabrication for industrial scalability, such as shortening mycelial incubation periods and automating environmental controls. Long-term performance assessments under realistic environmental conditions, comprehensive biodegradability analyses (including soil burial and enzymatic degradation tests to quantify breakdown rates), and detailed life-cycle assessments (LCAs) are crucial to further establish these composites as viable, sustainable alternatives. Cost analyses and pilot-scale fabrication will also be vital for evaluating their feasibility, particularly within the packaging and construction sectors.

## CRediT authorship contribution statement

**Pattaravaree Petchsong:** Conceptualization. **Nattawut Boonyuen:** Investigation. **Papichaya Kwantong:** Formal analysis. **Salilaporn Nuankaew:** Formal analysis. **Charuwan Chuaseeharonnachai:** Formal analysis. **Sarute Ummartyotin:** Writing – review & editing, Writing – original draft.

## Declaration of competing interest

The authors declare that they have no known competing financial interests or personal relationships that could have appeared to influence the work reported in this paper.
