# Supplementary material for: Development of mycelium-reinforced dialdehyde cellulose–polyvinyl alcohol composites for emerging high-performance sustainable materials
Source: Synth Syst Biotechnol. 2026 Mar 27;13:545–58. doi: 10.1016/j.synbio.2026.03.012 (PMC13054041; doi:10.1016/j.synbio.2026.03.012)
Supplement: Multimedia component 1 [file mmc1.docx]

**Supporting information**

**Supporting information 1 : Preparation of tensile testing**

For mechanical properties testing, samples were cut to 10 mm x 10 mm size, but the thicknesses varied because the thickness could not be controlled when the samples were put in an oven to stop mycelium growth.

**Supporting information 2: Table S1. The details of the mechanical properties.**

| **Composite materials** | **Sample No.1** | | | **Sample No.2** | | | **Sample No.3** | | | **Everage value** | | |
| --- | --- | --- | --- | --- | --- | --- | --- | --- | --- | --- | --- | --- |
|  | **Thickness** | **Tensile strength** | **Elongation** | **Thickness** | **Tensile strength** | **Elongation** | **Thickness** | **Tensile strength** | **Elongation** | **Thickness** | **Tensile strength** | **Elongation** |
| 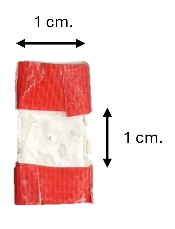  PVA/DAC | 5.26 mm. | 0.475 MPa | 30% | 5.58 mm. | 0.597  MPa | 30% | 5.70 mm. | 0.641 MPa | 40  % | 5.513  ±  0.23  mm. | 0.562  ±  0.14  MPa | 33.333  ±  5.77  % |
| 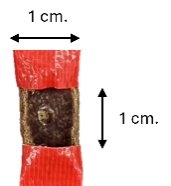  PVA/DAC/PDB | 2.25 mm. | 7.19  MPa | 200% | 2.19 mm. | 7.08 MPa | 200% | 2.09 mm. | 7.26 MPa | 230% | 2.177  ±  0.08  mm. | 7.177  ±  0.90  MPa | 210.000  ±  17.32  % |
| 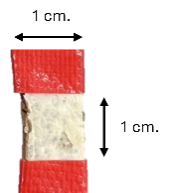  PVA/DAC/E | 1.03 mm. | 26.9 MPa | 20% | 1.42 mm. | 16.2 MPa | 50% | 1.21 mm. | 11.9 MPa | 30% | 1.220  ±  0.20  mm. | 18.333  ±  7.72  MPa | 33.333  ±  15.28  % |
| 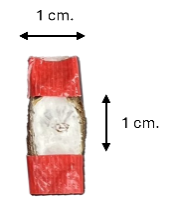  PVA/DAC/L | 2.29 mm. | 8.59 MPa | 110% | 2.24 mm. | 7.44 MPa | 90% | 2.30 mm. | 8.48 MPa | 80% | 2.277  ±  0.03  mm | 8.170  ±  0.63  MPa | 93.333  ±  15.28  % |
| 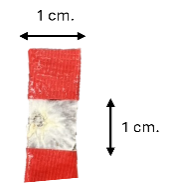  PVA/DAC/O | 1.51 mm. | 9.49 MPa | 60% | 1.22 mm. | 13 MPa | 90% | 1.09 mm. | 9.17 | 70% | 1.273  ±  0.22  mm | 10.553  ±  2.12  MPa | 73.333  ±  15.28  % |

**Supporting information 3: Figure S1 TGA thermograms of polyvinyl alcohol (PVA) and dialdehyde cellulose (DAC)**





**Supporting information 4: Figure S2 DSC thermograms of polyvinyl alcohol (PVA) and dialdehyde cellulose (DAC)**
